# A systematic review culturally adapted Cognitive Behavioral Therapy (CaCBT) for Arabic-speaking populations including North African subgroups

**DOI:** 10.1186/s40359-026-05125-w

**Published:** 2026-08-07

**Authors:** Rania Driouach, Constantina Badea, Sylvia Martin

**Affiliations:** 1 Université Paris Nanterre, Nanterre, France; 2 Center for Research Ethics and Bioethics, Faculty of Medicine and Pharmacy, Uppsala University, Uppsala, Sweden

**Keywords:** Culturally adapted cognitive behavioral therapy, Cognitive behavioral therapy, Arabic-Speaking populations, North African populations, Systematic review, Mental health, Cultural adaptation, Immigration, Acculturation

## Abstract

**Background:**

Cognitive behavioral therapy (CBT) is an effective treatment for common mental disorders. However, most CBT protocols have been developed within Western cultural frameworks, which may limit their relevance for culturally diverse populations. Culturally adapted CBT (CaCBT) seeks to enhance treatment acceptability and effectiveness by integrating linguistic, cultural, religious, and contextual factors. Despite growing interest in culturally adapted interventions, evidence regarding Arabic-speaking populations, including North African subgroups, remains fragmented.

**Methods:**

Following PRISMA 2020 guidelines, a systematic search was conducted in Cochrane Library, PubMed, PsycInfo, Scopus, and OpenAlex from inception to June 2025. Empirical studies assessing CaCBT among among Arabic-speaking populations, including North African subgroups in Europe or North Africa were included. Study selection involved independent double screening, and findings were synthesized narratively.

**Results:**

Thirteen studies met the inclusion criteria, including randomized controlled trials, qualitative studies, case reports, an exploratory dissemination study, and two review articles. CaCBT was associated with reductions in depression, anxiety, and post-traumatic stress disorder symptoms, particularly in internet-based formats. Common adaptations included language, culturally relevant metaphors, social norms, and occasionally religious or spiritual elements. High attrition rates were frequently reported in digital interventions.

**Conclusion:**

The available evidence suggests that culturally adapted CBT may be a promising intervention for Arabic-speaking populations, including North African subgroups. However, conclusions should be interpreted cautiously given the methodological heterogeneity of the evidence base, the predominance of studies involving broader Arabic-speaking populations, high attrition rates in digital interventions, and the limited reporting of participants’ countries of origin and migration-related characteristics. Future research should employ more rigorous designs, recruit more diverse and representative samples, and improve the reporting of cultural adaptation processes to strengthen the evidence base and inform culturally responsive mental health care.

**Supplementary Information:**

The online version contains supplementary material available at 10.1186/s40359-026-05125-w.

## Introduction

Cognitive Behavioral Therapy (CBT) is currently regarded as one of the most empirically validated psychotherapeutic approaches for the treatment of a wide range of mental disorders, including depression, anxiety disorders, and post-traumatic stress disorder [1–4]. Based on the principle that thoughts, emotions, and behaviors are interconnected, CBT aims to identify and modify dysfunctional cognitive patterns in order to improve patients’ overall psychological functioning [5].

However, the majority of CBT protocols have been developed within Western contexts, primarily in North America and Europe, and often reflect the individualistic and rationalist values predominant in these societies [6, 7]. This cultural grounding may limit their effectiveness when applied to populations from different cultural backgrounds, particularly immigrant and refugee populations [8, 9]. These groups often experience higher levels of psychological distress than the general population due to factors such as discrimination, socioeconomic insecurity, and migration-related trauma [10–12].

In response to these challenges, culturally adapted Cognitive Behavioral Therapy (CaCBT) has been developed to tailor therapeutic protocols to patients’ cultural values, beliefs, and practices. Such adaptations may involve language, metaphors, cultural references, or the structure of therapy sessions [7, 13]. Several studies have demonstrated the effectiveness of CaCBT in reducing depressive and anxiety symptoms among various immigrant and refugee populations, including Asian, Latin American, and Arabic-speaking patients [14–16]. These interventions have also been associated with significant improvements in post-traumatic symptomatology and enhanced treatment adherence when cultural dimensions are adequately addressed [9].

Nevertheless, although CaCBT has received growing attention within Arabic-speaking populations, evidence remains fragmented regarding North African populations, despite their substantial presence in Europe and their heightened exposure to psychological distress associated with migration, discrimination, and socioeconomic adversity [17, 18]. While Arabic-speaking populations are culturally heterogeneous, individuals from Morocco, Algeria, and Tunisia share several linguistic, religious, and cultural characteristics with other Arab groups, including the centrality of family networks, collective values, and the influence of Islam on health-related beliefs and practices. These cultural dimensions may shape the expression of psychological distress, help-seeking behaviors, and engagement with psychotherapy [19–21]. Therefore, investigating CaCBT across Arabic-speaking populations, while specifically considering the representation of North African subgroups, may contribute to a more nuanced understanding of culturally responsive psychological interventions in Arab contexts.

Objectives

### Objectives

The present systematic review aims to synthesize the available evidence on culturally adapted Cognitive Behavioral Therapy (CaCBT) interventions for Arabic-speaking populations, including North African subgroups. Specifically, the review seeks to (1) evaluate the reported effectiveness of CaCBT interventions across mental health outcomes and (2) identify the principal cultural adaptation strategies implemented within these interventions.

## Methods

### Review design and protocol

This systematic review was conducted in accordance with the PRISMA 2020 (Preferred Reporting Items for Systematic Reviews and Meta-Analyses) guidelines. We aimed to identify, analyze, and synthesize studies evaluating culturally adapted Cognitive Behavioral Therapy (CaCBT) interventions for North African populations residing in Europe and in North Africa.

### Literature search strategy

**Fig. 1:**
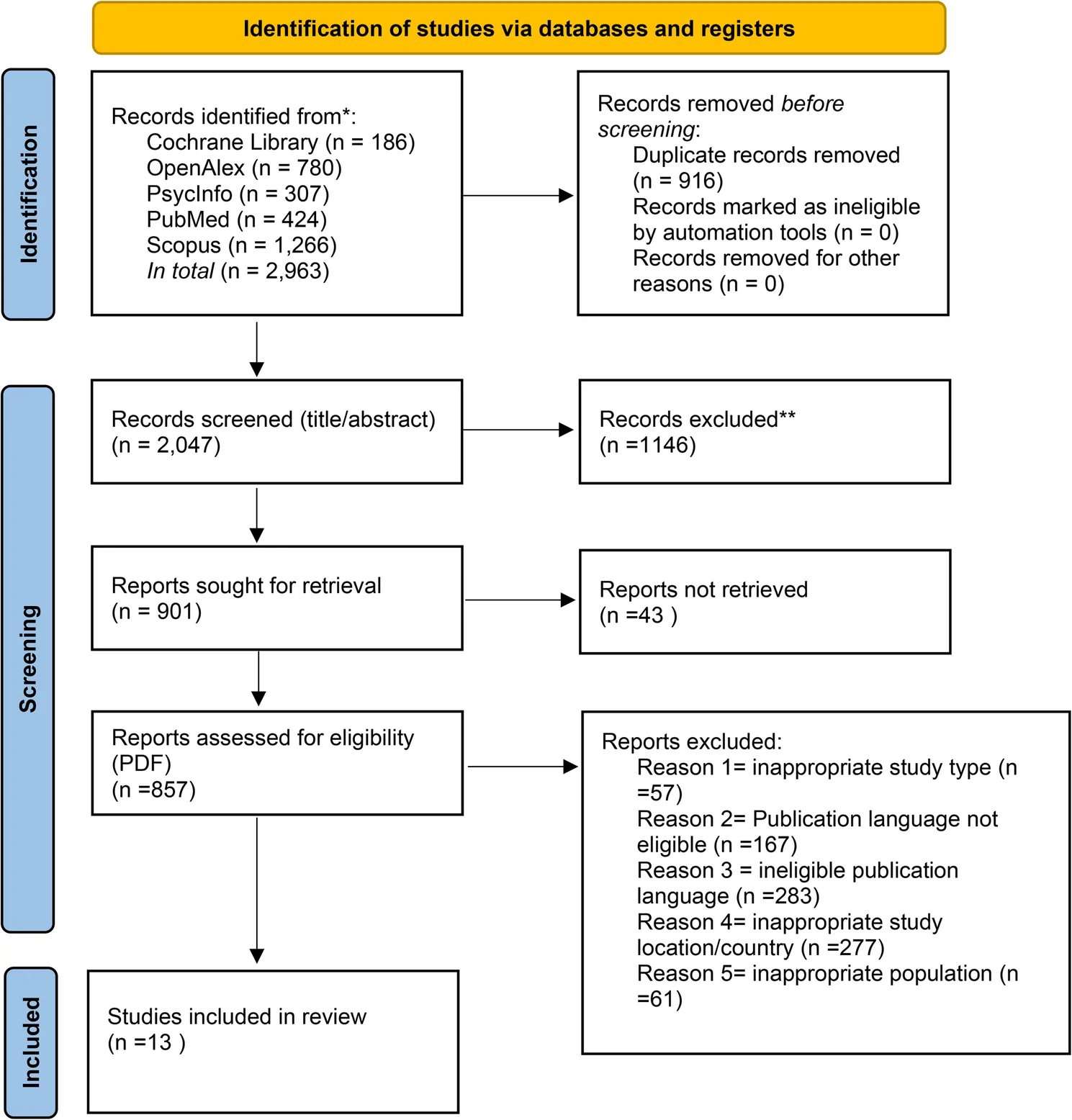
PRISMA chart Note:* Consider, if feasible to do so, reporting the number of records identified from each database or register searched (rather than the total number across all databases/registers). * * If automation tools were used, indicate how many records were excluded by a human and how many were excluded by automation tools The vertical diagram summarizes the identification, screening, eligibility, and inclusion process, where 2,963 records were identified, 2,047 screened after duplicate removal, and 858 full-text articles assessed, resulting in 13 studies included following predefined exclusion criteria

The search strategy combined keywords and Boolean operators related to CBT, cultural adaptation, and North African populations (example : (“North African People“[MeSH Terms] OR (“algeria*“[Title/Abstract] […]AND (“Cognitive Behavioral Therapy“[MeSH Terms] OR ((“Psychotherapy“[MeSH Terms] OR “mental disorders/therapy“[MeSH Terms]) […] OR “culturally therapy“[Title/Abstract:~3] OR “ethnically therapy“[ Title/Abstract:~3] OR “acceptance commitment“[Title/Abstract:~2]). The full search strategy and key terms used is available as (Supplementary file 2). The literature search was conducted without date restrictions, covering all records available from database inception to June 2025. The search was registered on PROSPERO CRD420251003415 (3 March 2025). It was conducted in Cochrane Library (*n* = 186), OpenAlex (*n* = 780), PsycInfo (*n* = 307), PubMed (*n* = 424), and Scopus (*n* = 1,266) scientific electronic databases (see Fig. 1). After merging records from all databases, 916 duplicate references were identified and removed. In addition, reference lists of included studies were manually screened to identify relevant publications not captured through the electronic search. A final set of 13 articles was selected for analysis (see Supplementary Material 1).

### Eligibility criteria

The inclusion criteria encompassed empirical studies that evaluated culturally adapted cognitive behavioral therapy (CaCBT) interventions. Eligible studies were required to involve participants from North African populations (countries), either residing in North Africa or within North African diaspora communities. In addition, included studies had to report at least one clinical or psychological outcome, such as symptoms of depression, anxiety, post-traumatic stress, or indicators of psychological well-being. Only publications written in English or French were considered for inclusion. In addition to primary studies, review articles were eligible when they provided relevant information regarding culturally adapted CBT interventions for Arabic-speaking populations and met all predefined inclusion criteria. Findings from review studies were interpreted separately from primary empirical evidence to avoid conflating different levels of evidence.

The exclusion criteria included non-empirical sources such as books, theses, clinical trial registries, conference abstracts, poster presentations, and conference posters. These sources were excluded because they frequently provided insufficient methodological detail regarding intervention content, cultural adaptation procedures, and outcome assessment. Studies were also excluded if the intervention was not at least partially based on cognitive behavioral therapy (CBT) or lacked a clear cultural adaptation component. Furthermore, research conducted on non-North African populations, or carried out in countries outside Europe and North Africa, was not eligible for inclusion in the review (see Table 1 for full description).

**Table 1:** Eligibility criteria

Description	Inclusion criteria
Population	Arabic-speaking individuals, including participants originating from North African countries (Morocco, Algeria, and Tunisia), whetherresiding in countries of origin, Europe, or other migration contexts. Studies involving broader Arabic-speaking populations were eligible when NorthAfrican participants were included or when country-specific data were not disaggregated
Testing	Effectiveness of Culturally Adapted Cognitive Behavioral Therapy (CACBT)
Comparators	Non-culturally adapted CBT
Outcomes	Effectiveness of the therapy
	Psychological distress (levels of anxiety, depression)
	Psychological well-being
	Anxiety
	Depression
	Emotional competencies
	Acculturation
Study design	Any
Language restrictions	English/French only
Publication type	Scientific article / review article / systematic review / case study
Country restrictions	Excluding studies conducted outside Europe and North Africa
Date restrictions	None

The table outlines the inclusion criteria regarding population (individuals of North African origin from Morocco, Algeria, and Tunisia), intervention (culturally adapted cognitive behavioral therapy), comparators (non-adapted CBT), and outcomes (psychological distress, well-being, anxiety, depression, emotional competencies, and acculturation). Additional criteria include study design (any), language (English or French), publication type (scientific and review articles), geographic scope (Europe and North Africa), and no date restrictions

### Study selection process

Identified references were exported to Rayyan website for duplicate removal and first round of double-blind study selection by RD, SM and CB. No AI tools were used. The initial extraction was performed by the University of Uppsala library team and uploaded to Rayyan by RD and SM for title and abstract screening. Discussions to resolve discrepancies regarding inclusion or exclusion decisions were conducted to reach a final decision among the three reviewers. Consensus was reached on 858 articles for full-text PDF double-blinded screening conducted independently by two reviewers (RD and SM), with 100% inter-reviewer agreement and no discrepancies requiring arbitration. Ultimately, 13 studies met the inclusion criteria and were selected for analysis (see Fig. 1). Scientific quality assessment was conducted using JBI tools, with scores ranging from 0.50 to 0.95 on a standardized scale (0–1), indicating an overall moderate-to-high methodological quality across the included studies. Quality assessment was conducted independently by two reviewers. Discrepancies were resolved through discussion. Quality ratings informed interpretation of findings but were not used as exclusion criteria. A detailed quality appraisal table has been provided in Table 2, including individual JBI item ratings, raw scores, and standardized quality scores for each study. Results of the quality appraisal are presented in Table 2. No studies were excluded based on quality assessment. All stages of the selection process are presented in the PRISMA flow diagram (Fig. 1).

**Table 2:**
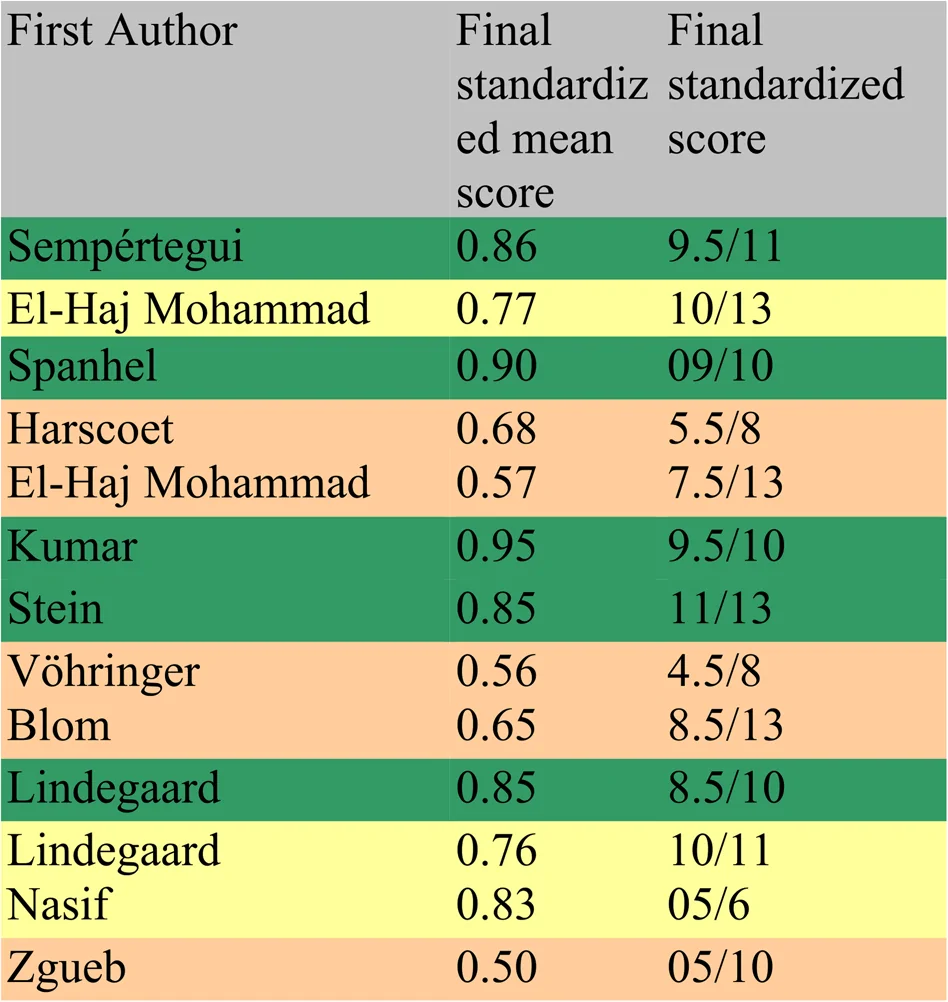
Methodological quality assessment of the included studies

Orange =0.50-0.69 →Moderate methodological quality; Yellow= 0.70-0.84 → Good methodological quality; Green =≥ 0.85 → Very good methodological quality. This table summarizes the quality evaluation of the studies included in the systematic review using standardized scores and corresponding methodological quality categories. Studies were classified according to their final standardized scores as showing moderate (0.50-0.69), good (0.700.84), or very good (≥0.85) methodological quality. The assessment highlights the variability in study rigor across the included articles, with most studies demonstrating good to very good methodological quality

### Data extraction and synthesis

Extracted data included the author(s) and year of publication, descriptive informations and study population characteristics. Details regarding the targeted mental disorder were also recorded and comprehensive information on intervention characteristics (see Figs. 3 and 5). Finally, the main outcomes were extracted, including measures of clinical effectiveness. A narrative thematic synthesis with an inductive approach was conducted to identify culturally adapted components across the included studies and to examine their reported associations with psychological outcomes, following established methodological guidelines for narrative synthesis in systematic reviews [22, 23].

To enhance conceptual consistency in the identification and classification of cultural adaptations, reported adaptation strategies were mapped onto the Ecological Validity Framework (EVF) proposed by [7]. This framework distinguishes eight dimensions of cultural adaptation: language, persons, metaphors, content, concepts, goals, methods, and context. The EVF was used as an interpretive framework during the narrative synthesis to facilitate comparison across studies.

## Results

### General characteristics of included studies

**Fig. 2:**
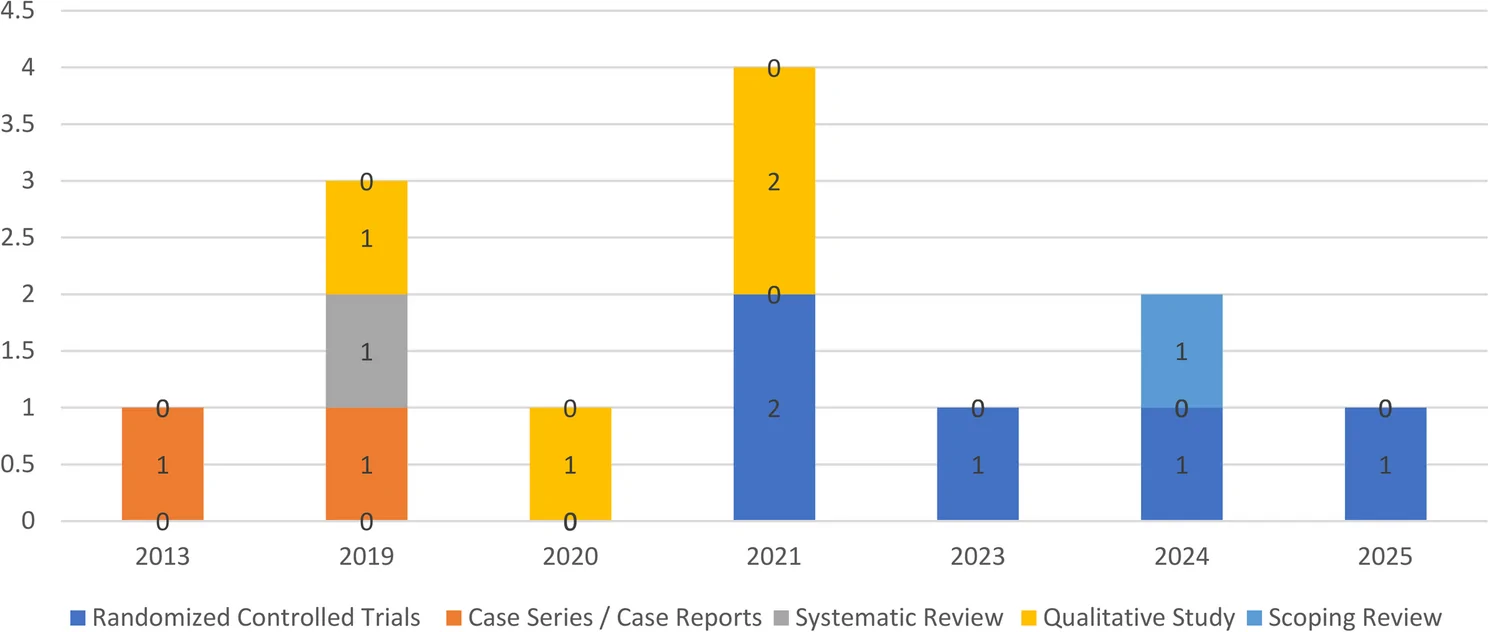
Types of articles per year The bar chart illustrates the temporal distribution of publications, showing peaks in 2019 and 2021, and a predominance of randomized controlled trials alongside qualitative studies and other designs

A total of 13 studies were included in the present systematic review (see Fig. 2). These studies were published between 2013 and 2025 and appeared in international peer-reviewed journals, primarily within the fields of psychiatry, clinical psychology [24–34] and digital mental health [35, 36].

### Study designs and levels of evidence

The 13 included studies employed heterogeneous methodological designs (see Fig. 2). Six studies were randomized controlled trials (RCTs) that compared online CBT interventions with wait-list control groups [25, 26, 29, 32, 33, 37], different modalities of online CBT (such as exposure versus cognitive restructuring) for example (see Fig. 5). Two studies consisted of literature-based designs [30, 31]. Two studies adopted an exploratory qualitative design [28, 35], while two others were case studies or case series [27, 34]. One study employed an open dissemination design and focused on predictors of attrition and treatment adherence within a culturally adapted internet-based CBT program rather than on treatment effectiveness outcomes [33]. This study was included because treatment engagement and retention constitute important dimensions of the acceptability and implementation of culturally adapted interventions.

### Data collection

#### Data sources and collection methods

The included studies relied on one or more data sources. Standardized self-report questionnaires were used in nine studies to assess outcomes such as depression, anxiety, post-traumatic stress disorder (PTSD), insomnia, and quality of life. Structured or semi-structured clinical interviews were conducted in eight studies [25, 26, 28, 33, 34, 36, 37], either in person, by telephone, or via online communication platforms. Qualitative data were collected through semi-structured interviews in four studies, reflective journals in one study, and think-aloud protocols in one study [28, 33, 35, 36]. In addition, two studies were literature-based and relied exclusively on published data [30, 31].

#### Contexts and settings of data collection

Data collection took place across a range of contexts. Eight studies collected data through secure online platforms [25–29, 32, 33, 37, 38]. Two studies were conducted in clinical settings [27, 34,] (see details in Supplementary Material 1). One study was conducted in a university setting [35]. Two literature-based studies did not involve direct data collection sites [31, 36].

### Study populations

#### Types of populations

**Fig. 3:**
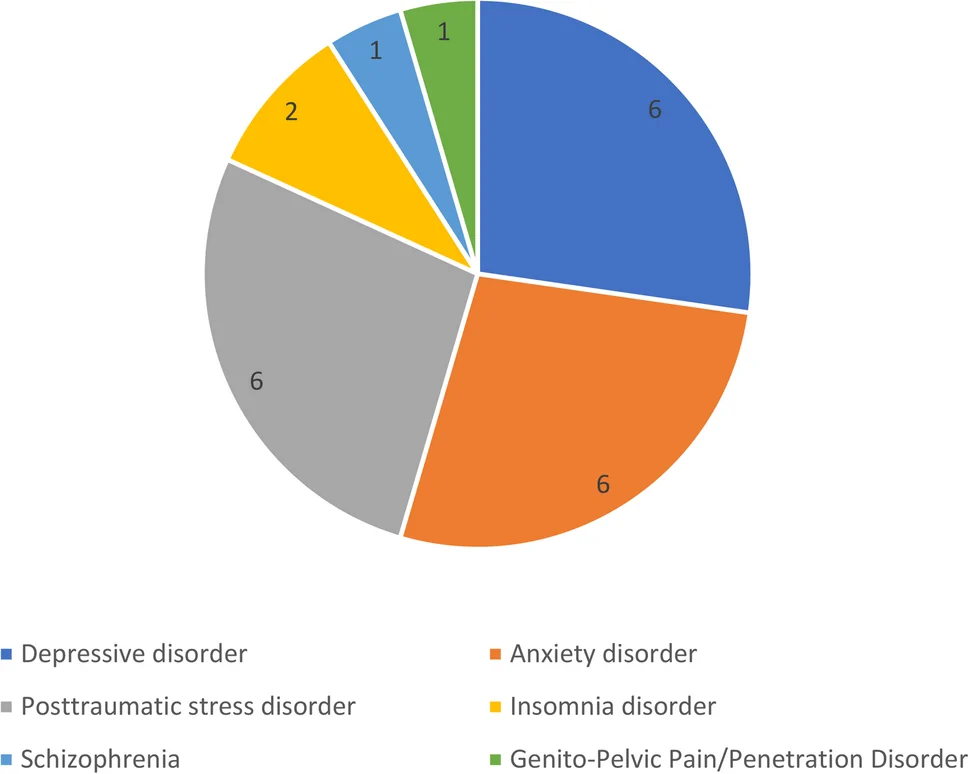
Distribution of therapeutic approaches across the included studies Depressive disorders, anxiety and post-traumatic stress disorder were the most frequently examined condition (n = 6 each). Insomnia disorder was assessed in two studies, while schizophrenia and genito-pelvic pain/penetration disorder were each addressed in a single study

Regarding study populations, eleven studies focused on individuals with a diagnosed mental disorder or clinically significant psychological symptoms [25, 34, 36, 37] (see Fig. 3). Two studies included mixed populations comprising patients, refugees, and/or healthcare professionals. One study focused exclusively on higher education students.

#### Samples demographics

Sample sizes varied according to study design ranges being from 11 to 743 participants. Case studies and case series involved between one and four participants. Most studies reported a predominance of female participants (*n* = 9), with proportions ranging from 58% to 75% in RCTs. Qualitative and clinical studies also showed a female majority, except for several studies conducted among predominantly male refugee populations (*n* = 3). Three studies did not specify gender distribution. Participants’ ages were mainly between 18 and 55 years, with mean ages ranging from 25 to 35 years in online interventions. One case study involved a 16-year-old adolescent, and one study did not report participant age.

#### Countries, participant origin, and migratory generation

**Fig. 4:**
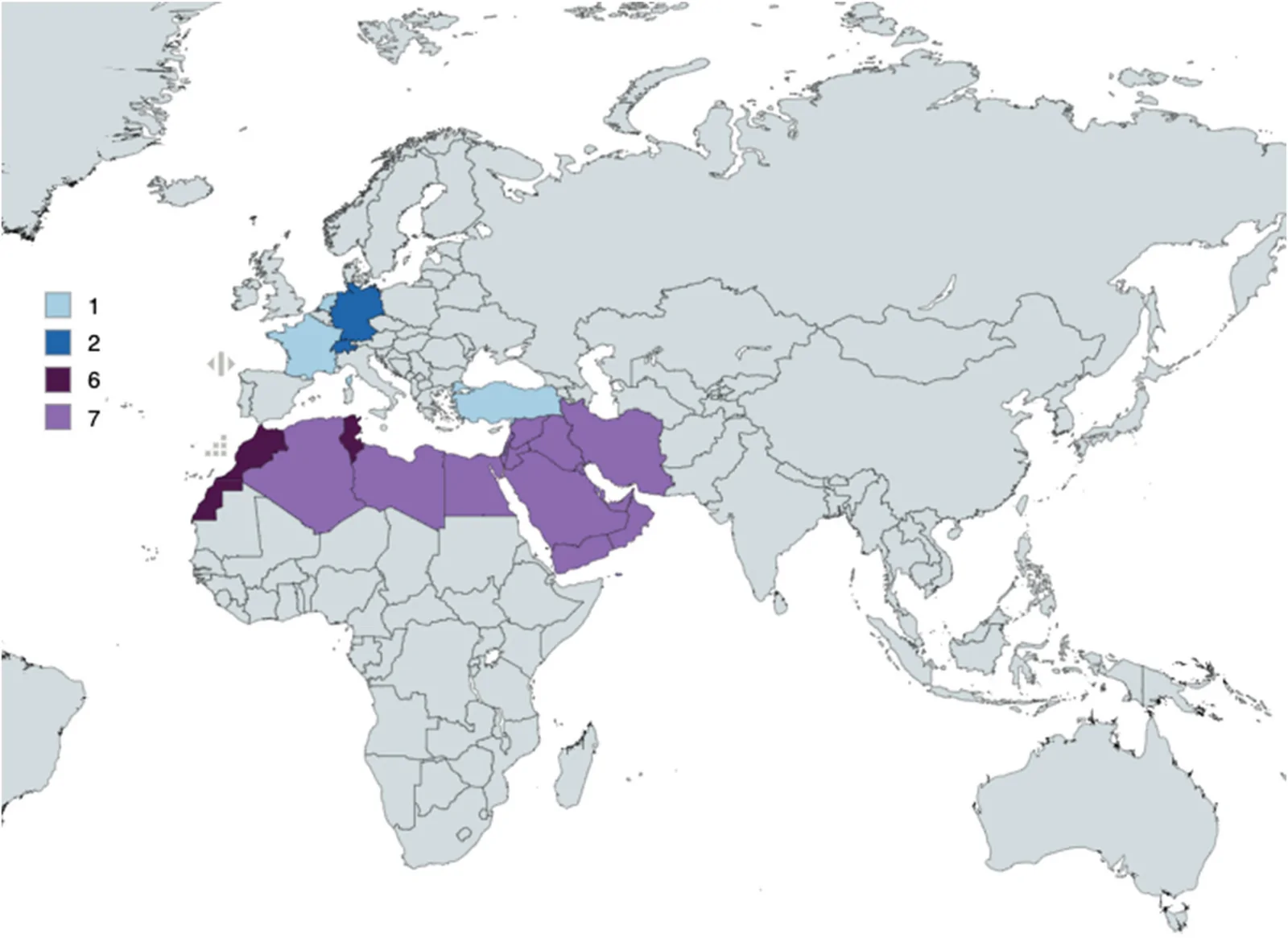
Geographical distribution of the studies Representation of participants’ countries of origin across included studies. Each count reflects the number of studies in which participants from a given country were included. Studies could contribute to multiple country categories; therefore, frequencies do not sum to the total number of included studies

The studies were conducted in Europe (*n* = 6) as well as in the Middle East and North Africa (MENA) region (*n* = 7). The latter included multi-country studies (*n* = 5) and studies conducted specifically in Morocco (*n* = 1) and Tunisia (*n* = 1) (See Fig. 4). Participants originated from a range of Arabic-speaking countries. Because several studies included individuals from multiple countries of origin, the frequencies presented in Fig. 4 reflect the number of studies in which a given country was represented rather than the total number of included studies.

Participants were predominantly Arabic-speaking [25–37], including individuals originating from North Africa [27, 30–32, 34] and other Arabic-speaking countries [25, 26, 28–30, 32–37]. Regarding migratory generation, four studies included exclusively first-generation [27–29, 36] one studie included both first- and second-generation participants [31], six studies did not report this information [25, 30, 32, 33, 35, 37], and two studies were conducted in countries of origin without a migration context [26, 34].

### Therapeutic interventions

#### Types of therapies and intervention formats

**Fig. 5:**
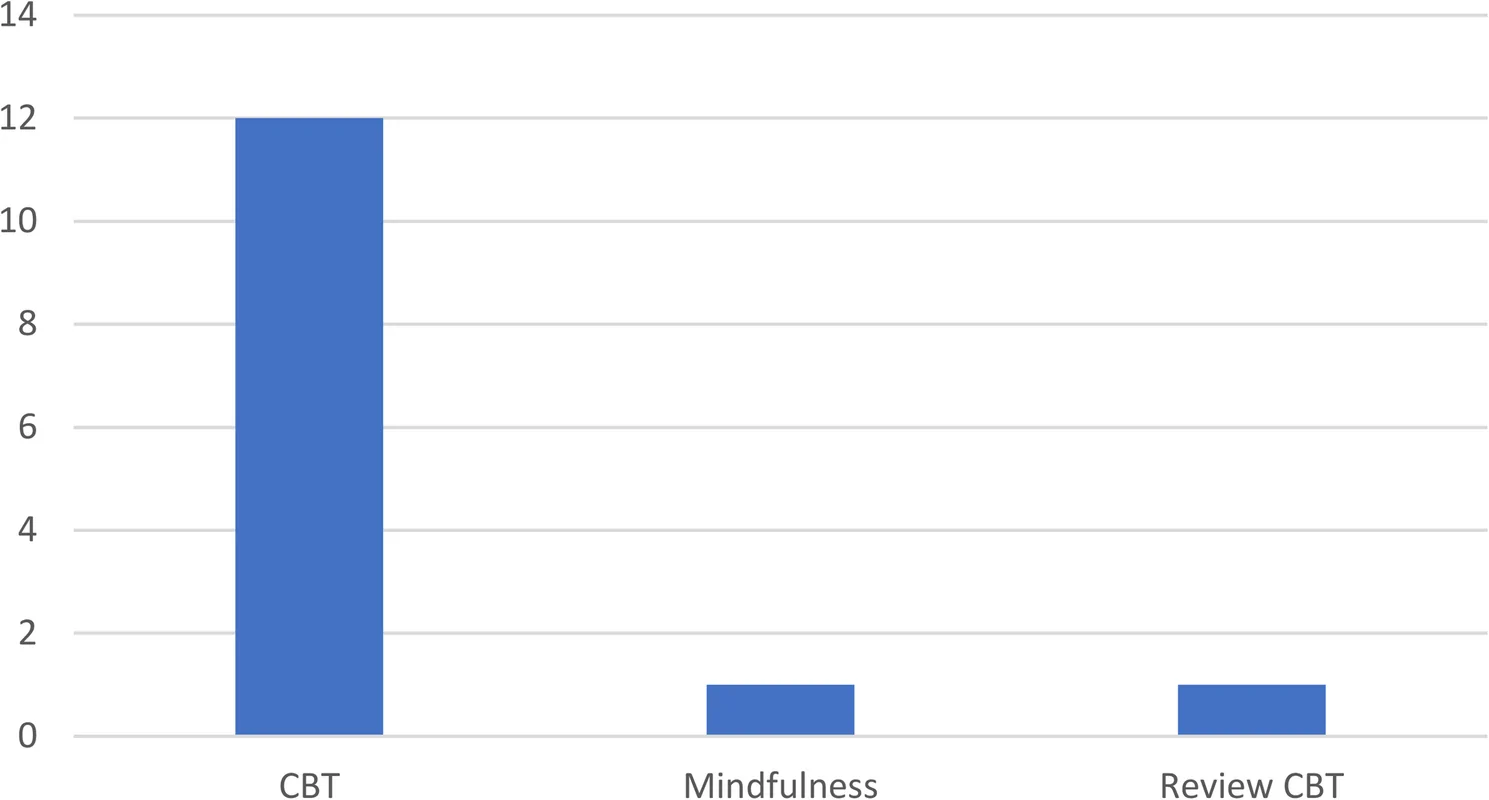
Distribution of therapeutic approaches across the included studies Cognitive behavioral therapy (CBT) was the predominant intervention, reported in 12 studies, mindfulness-based approaches were each examined in one study. Additionally, one study consisted of a review focusing on CBT

Twelve of the included studies examined Cognitive Behavioral Therapy (CBT) interventions [25–34, 36, 37]. These interventions were delivered through online formats (iCBT) in eight studies [25, 26, 28, 32, 33, 36–38], individual face-to-face formats in two studies [29, 30], and group formats, with or without interpreters, in one study [38]. One study assessed a culturally adapted mindfulness-based intervention [35], and one review synthesized various CBT formats [31] (See Fig. 5).

Online interventions primarily consisted of psychoeducation modules, therapeutic writing exercises, cognitive restructuring, imaginal exposure, behavioral activation, and relapse prevention components (see Supplementart Material 1. for details).

#### Cultural adaptations according to the ecological validity framework

The cultural adaptations reported across the included studies could be mapped onto several dimensions of the Ecological Validity Framework (EVF) proposed by [7]. Overall, adaptations primarily targeted language, content, concepts, methods, and context, whereas fewer studies explicitly addressed goals or deeper therapeutic processes.

Language adaptations were the most frequently reported dimension (*n* = 11). These adaptations included translation of intervention materials into Modern Standard Arabic, the development of gender-sensitive language versions, and the delivery of interventions by Arabic-speaking therapists or counselors. Several studies also reported the use of interpreters to facilitate communication and improve accessibility.

Persons and methods adaptations were reflected in modifications to the delivery of interventions, including the involvement of culturally competent clinicians, bilingual therapists, and interpreters. Some studies adapted the therapeutic format itself, for example through online delivery modes designed to increase accessibility among refugee and migrant populations.

Metaphors and content adaptations were commonly implemented through the incorporation of culturally relevant examples, narratives, and references. Several interventions integrated themes related to family relationships, collectivist values, honor, shame, gender roles, and parental authority. Visual materials and case examples were also modified to reflect participants’ sociocultural backgrounds.

Concepts adaptations involved the integration of culturally meaningful understandings of psychological distress and coping. In particular, several studies incorporated religious and spiritual dimensions, including references to Islam, prayer practices, Qur’anic teachings, or consultation with religious authorities. These adaptations aimed to increase the cultural acceptability of psychological interventions and align therapeutic content with participants’ belief systems.

Context adaptations were reported in six studies and addressed migration-related experiences, forced displacement, social exclusion, discrimination, socioeconomic adversity, and refugee status. These adaptations acknowledged the broader social and environmental factors influencing psychological distress and treatment engagement among Arabic-speaking populations.

Overall, the findings suggest that cultural adaptations were primarily focused on language and culturally relevant content, whereas fewer studies explicitly reported adaptations targeting therapeutic goals or core psychological mechanisms. This pattern indicates that most interventions emphasized improving cultural relevance and acceptability while preserving the fundamental principles of CBT.

#### Measurement of acculturation

Explicit measurement of acculturation was reported in only one study [31], which assessed cultural orientation, language proficiency, and acculturation strategies. The remaining 12 studies did not report any standardized measure of acculturation.

### Disorders targeted and clinical outcomes

Targeted conditions included depression (*n* = 6), anxiety disorders (*n* = 2), post-traumatic stress disorder (PTSD) (*n* = 4), insomnia (*n* = 1), vaginismus (*n* = 1), as well as well-being dimensions without a formal clinical diagnosis (*n* = 1) (see Fig. 3).

#### Primary outcomes

Among the five studies specifically targeting depression, four studies reported a statistically significant reduction in depressive symptoms following the intervention. These improvements were primarily observed in RCTs evaluating online or blended CBT interventions. In contrast, one study reported more mixed findings, failing to demonstrate specific intervention effectiveness among participants of Moroccan origin, suggesting potential heterogeneity of effects across cultural subgroups.

Regarding anxiety disorders, two studies reported a significant reduction in anxiety levels following intervention. Observed effects included not only symptom reduction but also improvements in social and occupational functioning, particularly in the context of social anxiety.

All four studies targeting PTSD reported a significant reduction in PTSD symptoms post-intervention. These improvements were consistently accompanied by reductions in comorbid symptoms, including depressive, anxiety, and somatoform symptoms.

No statistically significant differences were observed between different online CBT modalities (e.g., exposure versus cognitive restructuring; longer versus shorter formats), suggesting overall comparable effectiveness across evaluated protocols.

The study focusing on insomnia identified several elements requiring specific cultural adaptation of online CBT interventions. However, no quantitative outcome data on sleep symptom change were reported, as the analysis was primarily qualitative and exploratory.

The study addressing vaginismus reported improvements in sexual functioning among all four patients included in the case series, accompanied by reductions in anxiety and depressive symptoms.

Additionally, the study evaluating a culturally adapted mindfulness intervention reported positive subjective changes, particularly in psychological well-being, self-awareness, and personal transformation, without formal diagnostic clinical assessment.

#### Secondary outcomes

Beyond primary clinical outcomes, several studies reported improvements in secondary outcomes. Six studies reported improvements in quality of life, five studies demonstrated increased perceived social support, four studies reported reductions in somatoform symptoms, and three studies described increased knowledge and more positive attitudes toward psychological care.

### Reported limitations and attritions rates

Regarding attrition, rates were reported in nine studies, primarily those involving online intervention formats. Dropout rates ranged from 37% to 60%, indicating overall high attrition. Attrition was higher in internet-based interventions compared to face-to-face formats. One study specifically examined predictors of dropout rather than clinical outcomes and identified marital status, perceived treatment credibility, and year of program enrollment as significant predictors of attrition [33]. These findings provide valuable information regarding treatment engagement and implementation challenges in culturally adapted internet-based CBT programs. Methodological limitations were discussed in the 1 studies, including small sample sizes, non-representative samples, high attrition rates, lack of long-term follow-up, exclusive reliance on self-report measures, and methodological heterogeneity.

Beyond symptom reduction, treatment adherence represents a critical component of intervention effectiveness in routine clinical practice. The study by [33], although not primarily designed to evaluate clinical outcomes, contributed important evidence regarding predictors of dropout in culturally adapted internet-based CBT. Given the high attrition rates observed across several included studies, understanding factors influencing treatment engagement is essential for optimizing the implementation and acceptability of CaCBT interventions among Arabic-speaking populations.

## Discussion

### Overall interpretation of the main findings

The findings of this systematic review indicate that culturally adapted Cognitive Behavioral Therapy (CaCBT) is generally effective in reducing symptoms of depression, anxiety, and post-traumatic stress disorder (PTSD) among North African populations. This observation is consistent with evidence from previous systematic reviews and randomized controlled trials conducted among refugee and immigrant populations, which have generally reported favorable clinical outcomes following culturally adapted CBT interventions [39, 40]. These findings are broadly consistent with previous reviews conducted among migrant and refugee populations. For example [39], reported that culturally adapted psychological interventions generally yielded better engagement and symptom improvement than non-adapted approaches, particularly when adaptations addressed language, cultural values, and explanatory models of distress. Similarly [40], highlighted the importance of tailoring psychological interventions to the cultural and contextual realities of displaced populations and emphasized the role of linguistic accessibility and culturally relevant examples in enhancing intervention acceptability.

The present review extends this literature by specifically examining Arabic-speaking populations, including North African subgroups, and by providing a detailed synthesis of the adaptation strategies used across interventions. While previous reviews largely focused on migrant and refugee populations from diverse cultural backgrounds, the current review highlights the predominance of linguistic and contextual adaptations within Arabic-speaking communities and identifies important gaps, particularly regarding the limited use of religious adaptations and the scarcity of studies conducted in European healthcare settings. Furthermore, our findings underscore the continued lack of evidence specifically targeting North African populations despite their substantial representation among migrant communities in Europe.

The most consistent effects were observed for PTSD, aligning with previous research demonstrating that culturally adapted CBT interventions lead to significant reductions in trauma-related symptoms among refugees exposed to multiple traumatic events [14, 41]. These findings suggest that cultural adaptation enhances the clinical relevance of CBT protocols without altering their core therapeutic mechanisms.

The results of this review are in line with the international literature showing that CaCBT is at least as effective as, and often more acceptable than, standard CBT for non-Western populations [42, 43].

More specifically, the linguistic, narrative, and religious adaptations identified in the included studies are similar to those reported in Arab and Muslim populations, where a biopsychospiritual conceptualization of mental distress is prevalent [44]. The integration of these dimensions appears to strengthen the therapeutic alliance and reduce treatment resistance, as documented in several qualitative and mixed-methods studies [45].

Several explanatory mechanisms may account for the observed effectiveness. First, linguistic adaptations reduce cognitive load and facilitate the appropriation of core CBT concepts, a factor identified as critical in interventions targeting refugee populations [40].

Second, the integration of religious and spiritual references may function as a source of meaning and emotional regulation, particularly in Muslim cultures where religion represents a central coping resource [46]. These adaptations may enhance treatment acceptability and reduce stigma associated with psychological care.

Finally, consideration of migration-related contextual factors (e.g., legal status, socioeconomic precarity, social isolation, discrimination) allows for a clearer distinction between psychopathological distress and normative reactions to adversity. This approach is consistent with ecological models of cultural adaptation [47].

Beyond cultural factors, postmigration stressors may also play an important role in shaping mental health outcomes and responses to treatment among Arabic-speaking migrants and refugees. Previous research has demonstrated that experiences of discrimination, legal uncertainty, insecure residency status, unemployment, socioeconomic hardship, family separation, and barriers to healthcare access are strongly associated with depression, anxiety, and post-traumatic stress symptoms in migrant populations [48–50]. While several included studies acknowledged migration-related experiences during the adaptation process, few explicitly examined postmigration stressors as potential moderators of treatment effectiveness. As a result, it remains unclear to what extent the observed benefits of CaCBT may vary according to participants’ postmigration circumstances. Future research should therefore incorporate standardized measures of postmigration stress to better understand how social and structural factors interact with culturally adapted psychological interventions.

Mapping the identified adaptations onto the Ecological Validity Framework (EVF) revealed that language, content, and contextual adaptations were the most consistently reported dimensions across studies. In contrast, adaptations related to therapeutic goals and underlying conceptual models of distress were less frequently described. This pattern is consistent with previous literature suggesting that culturally adapted interventions often prioritize surface-level adaptations, such as language modification and culturally relevant examples, whereas deeper adaptations addressing culturally embedded explanatory models, values, and meanings of distress remain less frequently implemented. The EVF highlights eight dimensions of cultural adaptation, including language, persons, metaphors, content, concepts, goals, methods, and context, emphasizing that effective adaptation requires consideration beyond linguistic translation alone [58–60]. Future research should provide more detailed reporting of adaptation processes to facilitate replication and improve understanding of which dimensions contribute most strongly to treatment effectiveness [51].

Although the overall findings are encouraging, the mechanisms through which cultural adaptation may influence treatment outcomes remain insufficiently understood. Most studies evaluated culturally adapted interventions as a whole and did not isolate the effects of individual adaptation components. Consequently, it remains unclear whether observed benefits are primarily attributable to linguistic adaptations, culturally relevant content, contextual modifications, therapist characteristics, therapeutic alliance, or other intervention-related factors. Previous research has emphasized that cultural adaptation is a multidimensional process, and that the specific contribution of individual adaptation components remains difficult to determine [52, 53]. Future research should therefore aim to identify the active ingredients of culturally adapted interventions and examine their relative contribution to treatment engagement, acceptability, and clinical outcomes.

### Methodological limitations of the included studies and implications for interpretation

Despite the generally favorable findings supporting CaCBT, several cross-cutting methodological limitations in the existing literature must be considered, as they may influence the interpretation, generalizability, and reproducibility of the observed effects.

First, a number of included studies do not provide sufficiently detailed descriptions of the nature, intensity, and level of cultural adaptations implemented. In several cases, adaptation is mentioned in broad terms, without a clear distinction between peripheral adaptations (e.g., language, examples, materials) and core adaptations affecting therapeutic mechanisms. The literature emphasizes that insufficient documentation of adaptation processes constitutes a major barrier to identifying the active components of CaCBT and limits the reproducibility of interventions across clinical and cultural contexts [39, 47]. This methodological shortcoming hinders comparative evaluation and the development of standardized cultural adaptation models.

In addition, migratory generation is often insufficiently reported, despite being a factor known to influence mental health representations, expectations regarding psychotherapy, and the therapeutic alliance. The lack of distinction between first-generation immigrants, later generations, and non-migrant populations prevents the examination of potential differential effects of CaCBT according to exposure to the host culture, as suggested by recent reviews on psychological interventions for refugee and migrant populations [40].

Similarly, the explicit measurement of acculturation is largely absent from the included studies. This omission is problematic, as acculturation is considered a central indicator of cultural fit and potential treatment effectiveness. Previous research has shown that acculturation level may moderate therapeutic engagement, acceptability of cognitive-behavioral techniques, and treatment response [54]. The absence of standardized acculturation measures therefore limits a nuanced understanding of the mechanisms through which cultural adaptation operates.

Moreover, several studies do not report participants’ specific countries of origin, sometimes grouping heterogeneous populations under broad categories such as “Arabic-speaking” or “MENA populations.” However, cultural, historical, and sociopolitical contexts specific to countries of origin are likely to influence explanatory models of distress, coping styles, and attitudes toward psychological care [44]. This lack of precision limits external validity and complicates the application of findings to specific North African subgroups.

Finally, although North African populations are highly represented in many European countries, the number of studies conducted specifically in European contexts remains limited. This geographic underrepresentation is consistent with findings from recent reviews highlighting a lack of empirical data tailored to European healthcare systems and institutional frameworks [55]. It restricts the generalizability of findings to European clinical settings and underscores the need for more contextually grounded research.

This issue is not only a limitation of the included studies but also reflects a broader challenge within the literature on culturally adapted psychological interventions for Arabic-speaking populations. Participants from diverse Arabic-speaking countries are frequently aggregated into broad categories such as “Arab,” “Arabic-speaking,” or “MENA,” with limited reporting of country-specific characteristics. Such approaches may overlook important cultural, social, and historical variations existing within these populations, as Arab societies are characterized by substantial diversity in sociocultural norms, religious practices, migration trajectories, and healthcare contexts [56, 57]. While such classifications may facilitate recruitment and analysis, they risk obscuring important within-group differences related to migration histories, sociocultural norms, religious practices, experiences of discrimination, and patterns of healthcare utilization. Consequently, the current evidence base remains insufficient to determine whether adaptations developed for broadly defined Arabic-speaking populations are equally relevant and effective for specific subgroups, including individuals from Morocco, Algeria, and Tunisia [30, 57].

At the same time, the inclusion of North African participants within broader Arabic-speaking samples remains clinically relevant, as these populations share several linguistic, cultural, and religious references that frequently constitute the basis of reported cultural adaptations. However, the limited identification and characterization of North African participants across studies highlights an important research gap. Previous reviews have emphasized that successful cultural adaptation requires a detailed understanding of the target population, including local cultural meanings, community characteristics, and contextual factors rather than relying solely on broad cultural categories [39, 57]. Future research should therefore move beyond broad cultural classifications and provide more detailed reporting of participants’ countries of origin, migration trajectories, and cultural backgrounds. Such efforts would facilitate the development and evaluation of culturally adapted interventions that are both sensitive to shared Arabic-speaking cultural dimensions and responsive to the specific needs of North African communities, particularly within European mental health services where these populations are substantially represented.

Overall, these methodological limitations suggest that, despite promising results, the effects of CaCBT should be interpreted with caution. They highlight the importance of future research adopting systematic and transparent reporting of cultural adaptations, integrating measures of acculturation and migratory generation, and providing precise characterization of participants’ countries of origin to enhance external validity, comparability, and reproducibility.

### Limitations and future research

Several limitations should be considered when interpreting the findings of this review. First, methodological heterogeneity was observed (e.g. study design, intervention format, conditions, outcome measures) limiting comparability across studies, precluded the conduct of meta-analytic analysis or calculation of standardized effect sizes.

Second, most studies relied primarily on self-report measures, which may be vulnerable to social desirability, recall, and reporting biases that can be particularly important in some Arabic-speaking cultural contexts, where stigma surrounding mental health can influence authentic disclosure.

Third, high attrition was common, raising concerns about adherence and generalizability, particularly for less engaged individuals or those facing greater barriers. Inconsistent reporting of adherence also limited analysis of factors influencing retention (e.g., cultural adaptations, therapist support).

Another limitation is the lack of assessment of acculturation and migration-related factors. Most studies did not use standardized measures of acculturation, cultural identity, religiosity, or post-migration stressors, limiting insight into how these variables influence engagement, acceptability, and outcomes. Future research should systematically assess these factors and examine their moderating effects.

Sample representativeness is also foressen as a limitation. Participants were mostly young adults and women, often recruited via universities, online platforms, or specialized healthcare services. This likely underrepresents older adults, men, individuals with lower education, and those with limited digital access, constraining generalizability and underscoring the need for more inclusive recruitment.

A further limitation concerns the review’s scope. Although intended to focus on North African populations, most evidence involved broader Arabic-speaking groups, with North African participants rarely identified separately. As a result, conclusions for North African populations only should be interpreted cautiously. This also highlights a key gap and the need for studies specifically targeting North African communities in both origin and diaspora settings.

Limiting the search to English and French publications is another potential limitation. Excluding Arabic-language studies may have led to missing relevant research from Arabic-speaking contexts. Future reviews should include Arabic sources to provide a more comprehensive synthesis of culturally adapted psychological interventions.

A notable share of the evidence comes from a single research program. Five studies stem from the Ilajnafsy project or closely related initiatives and involve overlapping teams [25, 26, 32, 33, 37]. Although they address different aims and samples, they use similar platforms and adaptation approaches, limiting diversity and independence. Replication by independent groups across varied healthcare settings is needed to strengthen confidence in the effectiveness and generalizability of culturally adapted CBT.

## Conclusion

This systematic review demonstrates that CaCBT constitutes an effective and relevant intervention for the treatment of several mental disorders among North African populations. The findings suggest that culturally adapted CBT interventions are associated with reductions in depressive, anxiety, and post-traumatic symptoms among Arabic-speaking populations. Across studies, linguistic, cultural, and contextual adaptations were commonly reported, whereas religious and spiritual adaptations were less frequently incorporated. The limited use of religious adaptations highlights an important area for future research, particularly given the potential relevance of spirituality within many Arabic-speaking communities [40]. By addressing a significant gap in the literature, this review provides novel insights into the cultural specificities of the North African context and underscores the necessity of designing culturally sensitive psychotherapeutic interventions. The findings suggest that cultural adaptation may contribute to the acceptability and effectiveness of CBT interventions among Arabic-speaking populations. However, considerable variability in the reporting and implementation of adaptation strategies across studies makes it difficult to determine which specific components are most strongly associated with treatment outcomes.

From both scientific and clinical perspectives, these findings argue for the systematic integration of cultural dimensions in the development and dissemination of CBT interventions, as well as for mental health policies that promote accessibility and cultural appropriateness of psychological care. Future research should prioritize rigorous randomized controlled trials with larger, more representative samples and standardized reporting of cultural adaptation procedures. There is also a need to systematically assess acculturation, religiosity, and post-migration stressors to better understand their influence on engagement, outcomes, and the effectiveness of cultural adaptations. In particular, efforts should focus on developing culturally tailored CaCBT interventions for North African populations. Greater attention to long-term outcomes, mechanisms of change, and factors affecting engagement and retention is warranted, including enhancing therapeutic support in digital interventions to reduce attrition. Qualitative research can further inform culturally responsive care by capturing lived experiences of Arabic-speaking service users. Finally, clinicians would benefit from specialized training in transcultural psychology and culturally adapted CBT to ensure sensitive and effective intervention delivery.

## Supplementary Material

Supplementary Material 1.

## Data Availability

No datasets were generated or analysed during the current study.
