# Supplementary material for: A systematic review culturally adapted Cognitive Behavioral Therapy (CaCBT) for Arabic-speaking populations including North African subgroups
Source: BMC Psychol. 2026 Aug 7;14:1215. doi: 10.1186/s40359-026-05125-w (PMC13488148; doi:10.1186/s40359-026-05125-w)
Supplement: Supplementary file 1 [file 40359_2026_5125_MOESM1_ESM.docx]

| First Author | Year | Study/ Research Type (hierarchy of evidence) 1. Systematic Reviews 2. Meta-Analysis 3. Randomized Controlled Trials (RCT) 4. Cohort Studies 5. Case-Control Studies 6. Case Series, Case Reports 7. Narrative Reviews, Editorials, Expert Opinion 8.a Explorative qualitative study 8.b Qualitative research study 8.c Scoping review of empirical studies | Study Design/ Method (short description) | Data Collection Source (if described) for example: health records/ electronic health records, registers, surveys, summary/ resume, interviews with HCPs, survey among medical students, etc. | Population Characteristics and Study Outcomes Population Type code : 1. General Public 2. HCPs 3. Patients 4. Parents  5. Relatives/ Caregivers 6.aRefugees and healthcare providers working with refugees 6.b Students 6.c immigrants/migrants abroad 7. N/A | Population size (n)   *number of participants who successfully completed the study | Gender ratio (% male, % female) | Age range  (if available) | Country/-es | participants' origin (Immigrant generation I, II or III) | ² CBT Type | ³ Cultural adaptation | Disease or condition reviewed in the article - please, specify | Results therapy | Overall Conclusion -  "Take-Home Message" (1 or 2-sentence summary) |
| --- | --- | --- | --- | --- | --- | --- | --- | --- | --- | --- | --- | --- | --- | --- | --- |
| Sempértegui ¹ | 2019 | 1. | Systematic review of 28 peer-reviewed studies (2000–2017) on depression symptom manifestation, treatment effectiveness, and obstacles/facilitators in Turkish and Moroccan immigrants in Europe. | Published peer-reviewed studies (databases: PsycINFO, MEDLINE, Science Direct, Web of Knowledge, Cochrane) | Studies conducted in Germany, the Netherlands, Austria, and Sweden (for treatment effectiveness), with additional countries included for symptom manifestation and barriers/facilitators; Population code: 3. Assessment of symptom manifestation, treatment effectiveness (with a focus on CBT and culturally adapted therapies), and barriers and facilitators to therapy. | Turkish: 11,533; Moroccan: 5,278; Natives: 303,212 | Turkish: 62.3% women; Moroccan: 62.1% women; Natives: 66.4% women | Adults (≥18 years); some studies on elderly subgroups | Germany, Netherlands, Austria, Sweden | Turkish and Moroccan immigrant(Mostly I and some II) | 2.b | 2 | Depression | Mixed/limited evidence: weak evidence of CBT effectiveness for Turkish immigrants, none for Moroccan immigrants; psychosomatic rehabilitation with bioenergetic therapy showed improvements; high attrition in internet-based interventions | Turkish and Moroccan immigrants with depression often present combined mood and somatic symptoms; available therapies show limited evidence of effectiveness, especially for Moroccans; culturally adapted interventions and addressing socioeconomic barriers are crucial. |
| El-Haj Mohammad | 2025 | 3. | Parallel-group randomized controlled trial comparing internet-based CBT (iCBT), internet-based interpersonal treatment (iIPT), and waitlist control | Online questionnaires, Structured Clinical Interview for DSM-5 Disorders (SCID-5-CV) via voice-over-IP | Conducted online via a secure platform with participants located in Arabic-speaking countries; Population code: 3. Primary outcome: depressive symptom severity (PHQ-9). Secondary outcomes: anxiety (GAD-7), quality of life (EUROHIS-QoL-8), somatoform symptoms (PHQ-15), and perceived social support (MSPSS). | 743 randomized participants (iCBT: 243, iIPT: 247, waitlist: 253) | Across pooled studies: ~65% female, 35% male (exact per study varied) | Mean age 25.8 years (SD 7.95); no specific range reported | Participants lived mainly in Egypt, Saudi Arabia, Jordan, Germany, Morocco, Iraq, Turkey, Algeria; origin primarily from Egypt, Saudi Arabia, Jordan, Syria, Iraq, Morocco, Algeria | Primarily Arabic-speaking countries (Egypt, Saudi Arabia, Jordan, Syria, Iraq, Morocco, Algeria)  (NR) | 1.a | 2 | Depression (often comorbid with anxiety/PTSD) | Both iCBT and iIPT significantly reduced depressive and anxiety symptoms, improved quality of life, and increased perceived social support compared to waitlist. No significant difference between iCBT and iIPT. | Internet-based CBT and interpersonal therapy are both effective for treating depression in Arabic-speaking countries and may help address mental health care gaps in these regions.iIPT reduced somatoform symptoms more than waitlist; iCBT increased perceived social support from friends; iIPT increased perceived social support from family |
| Spanhel | 2019 | 8.a | Qualitative exploration of user experiences with a culturally adapted internet-based CBT intervention for insomnia (eSano Sleep-e) in refugees | Think Aloud sessions, semi-structured interviews | University of Freiburg Institute of Psychology, refugee workplaces, or familiar environments; Population code: 6.a. Identification of internet-based CBT components requiring cultural adaptation for refugee populations. | 12 participants (6 refugees, 6 healthcare providers) | Refugees: 1 female / 5 male; Healthcare providers: 4 female / 2 male | Refugees: 20–52 (M=38.0, SD=11.75); Healthcare providers: 27–66 (M=45.67, SD=14.50) | Germany (study site) | Syria, Iran, Eritrea, Algeria, Iraq.  (I). | 1.e | 3 | Insomnia (sleep problems) | Identified multiple elements requiring cultural adaptation to improve identification, acceptability, and engagement for refugees | Culturally adapting internet-based CBT for insomnia to refugees’ unique experiences, habits, and beliefs enhances intervention relevance and acceptance, despite diverse cultural backgrounds |
| Harscoet | 2013 | 6. | Single clinical case study describing culturally adapted CBT for a migrant adolescent with social anxiety disorder. | Clinical observations, therapy session notes, psychological assessments (e.g., Liebowitz Social Anxiety Scale). | Therapy sessions conducted in a clinical psychology setting in France; Population code: 3. Reduction of social anxiety symptoms and improvement in social cognition and interpersonal skills. | 1 | 100% female | 16 years old | France (host country) | Algeria.  (I) | 2.a | 3 | Social Anxiety Disorder (SAD) | Significant reduction in social anxiety severity (Liebowitz scale score decreased from severe to moderate); improved eye contact, verbal expression, and participation in social/professional situations; able to enroll in vocational training. | Culturally adapted CBT effectively reduced social anxiety symptoms in a migrant adolescent by addressing both cultural and migration-related factors alongside traditional CBT techniques. |
| El-Haj Mohammad | 2024 | 3. | Randomized controlled trial comparing an internet‐based, counsellor‐guided cognitive behavioural writing intervention (treatment group) to a waitlist control group. | Online self‐report questionnaires (BDI-II, HSCL-25, EUROHIS-QoL-8) and clinical interviews (CIDI) conducted via telephone or voice-over-IP. | Conducted remotely via an online platform, with counsellors based in Germany and Egypt; Population code: 3. Primary outcome: depressive symptoms. Secondary outcomes: anxiety symptoms and quality of life. | 259 participants (TG: 128, WG: 131) | 64.5% female, 35.5% male | 18–55 years (Mean = 25.78, SD = 6.39) | Participants originated mainly from Egypt, Saudi Arabia, Morocco, Algeria, Syria, and Palestine; residing mostly in Egypt, Saudi Arabia, Morocco, Algeria, and Jordan. | Arabic-speaking countries in the MENA region (including Maghreb countries).  (Non-immigrant, majority); 18.9% abroad (NR)) | 1.c | 2 | Depression (with comorbid anxiety symptoms assessed). | Significant reduction in depressive symptoms (medium-large effect), improved QoL (medium effect), and reduced anxiety symptoms in completers (medium effect). | The culturally adapted, counsellor-guided iCBT was feasible and effective in reducing depression and improving QoL among Arabic-speaking adults in the MENA region. |
| Kumar | 2021 | 8.b | Interpretive phenomenological analysis of students’ experiences in an academic mindfulness course | Weekly critical reflective journals; semi-structured interviews | Al Akhawayn University, Ifrane, Morocco; Population code: 6.b. Exploration of students’ experiences and meaning-making processes related to participation in a mindfulness course, including self-awareness, well-being, compassion, and personal transformation. | 12 students (out of 17 enrolled) completed both journals and interviews | Not specified | Not specified | Morocco | one Lebanese, one Libyan, 13 Moroccans, and two Americans.  (NR) | 3.a | 4 | N/A – focus on personal transformation and well-being | Increased self-awareness, improved well-being, greater compassion, ability to pause in crises, perspective-taking, recognition of personal change | A culturally and spiritually adapted mindfulness course in a Moroccan university fostered profound personal transformation, well-being, and self-awareness in students. Connections to faith and prayer; improved relationships; reduced conflict; enhanced academic coping |
| Stein | 2023 | 3. | Three-arm randomized controlled trial comparing two brief internet-based CBT interventions (exposure vs. cognitive restructuring) with a waitlist control in Arabic-speaking participants with PTSD. | Self-report questionnaires (online) and structured clinical interviews (telephone or VoIP). | Online platform (Ilajnafsy), with participants recruited from the Middle East and North Africa and residing in multiple countries; Population code: 3. Primary outcome: change in PTSD symptom severity (PCL-5). Secondary outcomes: posttraumatic maladaptive beliefs, trauma-related guilt, anxiety (GAD-7), depression (PHQ-9), somatoform symptoms (PHQ-15), quality of life (EUROHIS-QoL-8), and treatment satisfaction. | 365 randomized (CR: 118, Exposure: 122, Waitlist: 125); 200 started treatment; 123 completed treatment. | 74.5% female, 25.5% male. | 18–53 years (mean = 25.49, SD = 6.68). | Participants from Egypt, Saudi Arabia, Syria, and other MENA countries; residing in Egypt, Saudi Arabia, Jordan, and others. | Middle East and North Africa (Arabic-speaking).  (NR) | 1.b | 3,4 | Posttraumatic Stress Disorder (PTSD). | Both treatments significantly reduced PTSD, anxiety, depression, somatoform symptoms, and maladaptive cognitions; improved quality of life; no significant difference between exposure and cognitive restructuring. | Brief internet-based CBT, whether exposure-focused or cognitive restructuring-focused, is effective and culturally adaptable for Arabic-speaking individuals with PTSD. High treatment satisfaction; similar dropout rates (~38-40%) in both active conditions. |
| Vöhringer | 2020 | 3. | Exploratory analysis using LASSO regression to identify predictors of dropout in internet-based CBT for PTSD | Online-administered questionnaires (socio-demographic, psychometric scales) + clinical interviews via phone/web | Conducted via an online platform, with therapists based in Egypt and Germany; Population code: 3. Examination of predictors of dropout, including marital status, treatment credibility, and year of registration. | 386 | 69.4% female, 30.6% male | 18–49 years (mean 26.0, SD 6.02) | Participants from 21 countries of origin; main: Egypt, Saudi Arabia, Syria, Algeria, Morocco; residing in 33 countries | Arabic-speaking, from MENA region.  (NR) | 1.b | 2 | Posttraumatic Stress Disorder (PTSD) | Dropout rate = 37.3%; three predictors identified (marital status, treatment credibility, year of registration) | Dropout prediction is challenging; identified predictors suggest focusing on divorced patients, improving treatment credibility, and updating technology to match usage patterns |
| Böttche | 2021 | 3. | Randomized controlled trial comparing two internet-based CBT protocols for PTSD in Arabic-speaking countries: TF-reg (10 assignments, 2 cognitive techniques) vs. TF-short (6 assignments, 1 cognitive technique). | Online self-report questionnaires; telephone/voice over IP-administered clinical interviews. | Conducted online with counsellors based in Egypt and Germany; Population code: 3. Primary outcome: PTSD symptom change measured by the Posttraumatic Stress Diagnostic Scale. Secondary outcomes: depression and anxiety (HSCL-25), somatic complaints (SCL-90-R somatic subscale), and quality of life (EUROHIS-QoL-8). | 224 | 67.4% female, 32.6% male | 17+ years; Mean = 25.3 years (SD = 5.9) | Netherlands. | Arabic-speaking countries (MENA region).  (NR) | 1.b | 1 | Posttraumatic Stress Disorder (PTSD) | Both TF-reg and TF-short significantly reduced PTSD symptoms (~15-point decrease on PDS) with no significant differences between groups; large within-group effect sizes; similar dropout rates. | A shorter internet-based CBT with one cognitive technique is as effective as a longer version with two techniques for reducing PTSD symptoms in Arabic-speaking populations in the MENA region, making it more scalable. Both interventions significantly improved depression, anxiety, somatic complaints, and quality of life. |
| Lindegaard | 2021 | 8.b | Semi-structured telephone interviews analyzed using Thematic Analysis | Semi-structured telephone interviews | Conducted via telephone with participants located in Sweden; Population code: 3. Exploration of participants’ experiences with culturally adapted internet-based CBT, including facilitators and barriers to treatment engagement. | 11 participants interviewed (subset of 59 in the original RCT) | 40% male, 60% female | 20–49 years (interviewed sample) | Sweden (host country) | Arabic-speaking immigrants and refugees (countries are not specified).  (I) | 1.d | 2 | Depression and Anxiety | Reported increased knowledge, self-awareness, positive attitude change toward help-seeking, but barriers included technical issues, difficulty applying material, and stressful life circumstances | Culturally adapted ICBT can reduce stigma, encourage further treatment-seeking, and be beneficial, though not all participants benefit equally; refugee-specific stressors should be addressed to improve engagement |
| Lindegaard | 2021 | 3. | Randomized controlled pilot trial comparing 8-week internet-based CBT (ICBT) with wait-list control | Self-report questionnaires (PHQ-9, GAD-7, ISI, PSS-14, IES-R, BBQ, AUDIT) and short clinical interviews | Conducted online via a secure treatment platform with recruitment through social media, search engine advertisements, and clinics targeting immigrant and refugee mental health; Population code: 3. Primary outcome: depressive symptoms (PHQ-9). Secondary outcomes: anxiety (GAD-7), perceived stress (PSS-14), insomnia (ISI), PTSD symptoms (IES-R), and quality of life (BBQ). | 59 randomized participants (30 ICBT, 29 wait-list) | 58% male, 42% female | 20–69 years | Sweden (host country) | Arabic-speaking immigrants and refugees (countries are not specified).  (I) | 1.d | 5 | Depression, anxiety, insomnia, stress, PTSD symptoms | Significant reduction in depressive symptoms (large effect size, d = 0.85), stress (d = 1.12), insomnia (d = 0.68); moderate but non-significant effect for anxiety; no significant effect for PTSD symptoms or quality of life | Culturally adapted ICBT can be an effective intervention for reducing depression, insomnia, and stress in Arabic-speaking immigrants/refugees in Sweden; larger trials needed. Improvement in some secondary outcomes; no reported negative effects. |
| Nasif | 2024 | 8.c | Scoping review following PRISMA-ScR framework, mapping cultural adaptation processes and components for CBT in Arab populations. | Published empirical peer-reviewed studies (databases: PubMed, CINAHL, Web of Science, Google). | Not applicable (literature-based systematic review); Population code: 6.c. Identification of cultural adaptation components, processes, and gaps in CBT interventions for Arab populations. | Not applicable for the review itself; 21 studies included (sample sizes vary per study). | Not specified. | Not specified. | Studies from Arab countries and countries hosting Arab immigrants (Europe, Australia, USA, etc.). | Arab (including Maghreb).  (NR) | 4.a | 5 | depression, anxiety, PTSD, schizophrenia, insomnia, general psychological distress. | Cultural adaptation improves acceptability and feasibility; tends to be superficial (content-focused) with limited contextual changes; positive outcomes reported in engagement and symptom reduction in several studies. | Cultural adaptation of CBT for Arab populations is essential and beneficial, but often limited to superficial changes; deeper contextual adaptation is needed to address cultural, religious, linguistic, and social factors. |
| Zgueb | 2019 | 6. | A case series of four Arab-Muslim women with vaginismus in Tunisia, highlighting cultural influences and adaptations of CBT treatment. | Clinical interviews, psychiatric assessments, Beck Depression Inventory II, State and Trait Anxiety Inventory. | Department of Psychiatry A, Razi Hospital La Manouba, Tunisia, and gynecology and obstetrics services in Tunis; Population code: 3. Reduction in anxiety, depression, and vaginismus symptoms, improvement in ability to engage in sexual intercourse, and enhancement of sexual desire when applicable. | 4 | 100% female | 25–35 years | Tunisia | Arab-Muslim, Maghreb region (Tunisia).  (Not immigrants, native Tunisians) | 5.a | 3, 4 | Vaginismus (Genito-Pelvic Pain/Penetration Disorder). | All four patients showed reduction in anxiety and/or depression scores, improved ability to have sexual intercourse; one case without sexual desire/orgasm but able to resume intercourse. | Vaginismus in Arab-Muslim women should be treated as a culturally influenced family issue; integrating cultural beliefs, family involvement, and religious perspectives into CBT can significantly improve treatment outcomes. Improved sexual desire in one case; enhanced family understanding of sexual health; education on women’s rights in marriage. |

**Table 1.** Included articles description.

¹Only this study assessed acculturation, which was measured through orientation toward the culture of origin versus the host culture, language proficiency, and acculturation strategies.

² CBT Type :

1a = Internet-based CBT (standard/adapted)

1b = Internet-based trauma-focused CBT

1c = Writing-based CBT

1d = Tailored ICBT

1e = CBT for insomnia

2a = Face-to-face adapted CBT

2b = Group CBT

3a = Mindfulness-based intervention

3b = Interpersonal Psychotherapy (IPT)

4a = Mixed CBT approaches

5a = CBT + family/systemic/religious

³ Cultural adaptation :

1 = Linguistic only

2 = Surface adaptation

3 = Deep adaptation

4 = Religious/spiritual integration

5 = Participatory structured adaptation

6 = No adaptation

The table summarizes key information for each study, including authorship, year, study type and design, data sources, population characteristics (size, gender distribution, age, origin, and setting), type of cognitive behavioral therapy and level of cultural adaptation, targeted conditions, main outcomes, and overall conclusions regarding the effectiveness and applicability of culturally adapted interventions.
